# Correlation between social functioning and quality of life in patients diagnosed with schizophrenia

**DOI:** 10.1192/j.eurpsy.2022.513

**Published:** 2022-09-01

**Authors:** B. Ristic, B. Batinic

**Affiliations:** 1 Institute of Mental health, Clinic For Addiction Treatment, Belgrade, Serbia; 2 Faculty of Philosophy, Department Of Psychology, Belgrade, Serbia; 3 University Clinical Centre of Serbia, Clinic Of Psychiatry, Belgrade, Serbia

**Keywords:** schizophrénia, social functioning, Quality of Life

## Abstract

**Introduction:**

Social dysfunction is a significant feature of schizophrenia leading to deminution of quality of life (QoL).

**Objectives:**

To explore the correlation between social functioning and quality of life in patients diagnosed with schizophrenia.

**Methods:**

The study sample comprised 32 patients diagnosed with schizophrenia (24 males and 8 females) recruited from the Clinic for mental disorders “Dr Laza Lazarevic” in Belgrade, with a mean age of 41.28 years (min 24, max 62), assessed by the Social Functioning Scale (SFS) and the World Health Organization Quality of Life questionnaire (WHOQOL-BREF).

**Results:**

There were two significant quasi-canonical positive correlations between social functioning and QoL (1: f-test=16.4, p=.001; 2: f-test=23, p=0.0001.) The first structure is formed through the set of SFS subscales- Recreation, Independence-performance and Independence-competence and set of QoL subscales- Mental health, Physical health, Environment and General assessment of QoL. The second structure is formed through the set of SFS subscales- Interpersonal functioning and Social engagement/withdrawal and the set of QoL subscales- General assessment of QoL and Environment. Furthermore, the first canonical component indicates a greater overlap of the opposition set by social functioning (23%) which leads to the assumption that the direction of influence goes from social functioning to QoL. Due to the equality of redundancy in the second canonical component, the direction of influence can only be inferred on the basis of the first canonical component.

**Conclusions:**

Social functioning and quality of life are related in patients diagnosed with schizophrenia, and this relationship is based on specific subfactors within those areas.

**Disclosure:**

No significant relationships.

